# Immune-Related Adverse Events (irAE) in Cancer Immune Checkpoint Inhibitors (ICI) and Survival Outcomes Correlation: To Rechallenge or Not?

**DOI:** 10.3390/cancers13050989

**Published:** 2021-02-27

**Authors:** Heidar J. Albandar, Jacob Fuqua, Jasim M. Albandar, Salahuddin Safi, Samuel A. Merrill, Patrick C. Ma

**Affiliations:** 1 West Virginia University Cancer Institute, Morgantown, WV 26506, USA; heidar.albandar@hsc.wvu.edu (H.J.A.); salahuddin.safi@hsc.wvu.edu (S.S.); samuel.merrill@hsc.wvu.edu (S.A.M.); 2 Department of Medicine, West Virginia University, Morgantown, WV 26506, USA; jacob.fuqua@hsc.wvu.edu; 3 Department of Periodontology and Oral Implantology, Temple University School of Dentistry, Philadelphia, PA 19140, USA; jasim.albandar@temple.edu; 4 Penn State Cancer Institute, Penn State College of Medicine, Penn State Health Milton S. Hershey Medical Center, Pennsylvania State University, Hershey, PA 17033, USA

**Keywords:** checkpoint inhibitor, immune related adverse event, rechallenge of immunotherapy

## Abstract

**Simple Summary:**

This study examined the real-world experience and occurrence of immune-related adverse events (irAEs) under cancer checkpoint immunotherapy, and the relationship between its treatment rechallenge status and their impact on clinical outcomes. The current study demonstrates that immune checkpoint inhibitors (ICI) reinitiation after an irAE-related interruption in the setting of cancer immunotherapy was not associated with significantly improved survival outcome when compared with those without ICI treatment reinitiation after irAE-related therapy interruption.

**Abstract:**

Introduction: There is growing recognition of immune related adverse events (irAEs) from immune checkpoint therapies being correlated with treatment outcomes in certain malignancies. There are currently limited data or consensus to guide management of irAEs with regards to treatment rechallenge. Methods: We conducted a retrospective analysis with an IRB-approved protocol of adult patients seen at the WVU Cancer Institute between 2011–2019 with a histopathologic diagnosis of active cancers and were treated with immune checkpoint inhibitors (ICI) therapy. Results: Demographics were similar between the ICI interrupted irAE groups within cancer types. Overall, out of 548 patients who received ICI reviewed, there were 133 cases of ≥1 irAE found of any grade. Being treated with anti-CTLA-4 inhibitor ICI was associated with lower risk of death compared to anti-PD-1 ICI. The overall survival difference observed for irAE positive patients, between rechallenged (37.8 months, reinitiated with/without interruption; 38.6 months, reinitiated after interruption) and interrupted/non-reinitiated (i.e., discontinued) groups (24.9 months) was not statistically significant, with a numerical trend favoring the former. Conclusions: Our exploratory study did not identify significantly different survival outcomes among the Appalachian West Virginia adult cancer patients treated with ICI who developed irAE and had treatment reinitiated after interruption, when compared with those not reinitiated.

## 1. Introduction

The adoption of immunotherapy has led to a paradigm shift in how clinicians view the treatment of advanced stage malignancies. In particular, survival outcomes have improved markedly for previously morbid advanced stage non-small cell lung cancer, melanoma, and other malignancies when treated with immune checkpoint inhibitors (ICIs) as a form of cancer immunotherapy [1,2]. There are 6 main programmed death (PD)-1/PD-L1/cytotoxic T-lymphocyte-associated protein 4 (CTLA-4) inhibitors in clinical practice that have been granted approval by the U.S. Food and Drug Administration (FDA) in recent years: pembrolizumab, nivolumab, atezolizumab, avelumab, durvalumab, and ipilimumab. Each of these ICIs has been approved in specific stages of specific malignancies with the exception of pembrolizumab; which has a tumor agnostic biomarker indication that can be met with microsatellite-instability (MSI)-high status. Studies investigating factors that predict immune-related adverse events (irAEs) have been limited to non-small cell lung cancer, melanoma, and renal cell carcinoma. Furthermore, they have been primarily limited to patients receiving ipilimumab, nivolumab, or pembrolizumab [3].

The primary response to T-dependent antigens during humoral immunity includes B-lymphocytes initiating a cascade of events that culminate in the circulation of many B-memory cells and T4-memory cells [4]. These circulating cells play a major role in the anamnestic response to future similar antigen exposure and does so, in part, by migrating to the bone marrow where they continue to secrete antibodies up to a period of months or even years after the last detected antigen has been destroyed [5]. This period of anamnestic immune response varies from patient to patient. As a result, there is potential benefit to prolonging the effects of immunotherapy in patients with irAEs by extending this finite period of anamnestic humoral immune response. As clinicians have gained more experience incorporating immunotherapy into clinical practice, observations have been made to the positive correlation between developing immune related adverse events and overall patient outcomes [6,7]. There are few studies examining whether there are significant changes in patient outcomes in cases where immunotherapy is resumed after an irAE [8]. Although it has been suggested that patients with cancer sustain durable responses from immunotherapy after overcoming these adverse events and not resuming therapy, there is insufficient longitudinal data to support this clinical practice. The possibility of the immunotherapy microenvironment “settling down” after several months or years cannot be readily discounted. Such a possibility could endanger any gains obtained from immunotherapy and lead to relapse of disease.

Checkpoint inhibitors are believed to improve survival outcomes in patients with metastatic non-small lung cancer and metastatic melanoma through a myriad of “revving up” the immune system to preferentially target tumor cells. A consequence of this mechanism of action, and a simple marker of patient response to immunotherapy, is a grade 1 through 4 irAE. There are various well described adverse events associated with immune checkpoint inhibitors involving largely all the internal organs [9]. IrAEs that require treatment with steroids and other immunomodulating therapies do not necessarily affect patient outcomes adversely [6,10]. Corticosteroids are a mainstay of American Society of Clinical Oncology (ASCO) and the National Comprehensive Cancer Network (NCCN) guidelines for managing a majority of irAEs [11]. Corticosteroids have anti-inflammatory and immunosuppressive effects that can interfere with the innate and adaptive immune system. As a result, patients treated with corticosteroids at doses equal to or higher than 10 mg/day of prednisone (or its equivalent) have been systematically excluded from immunotherapy clinical trials.

There are significant disparities in cancer incidence and mortality in the Appalachian West Virginia state in comparison to the rest of the nation. In this single center cohort study, our objective was to investigate the patient characteristics and outcomes who developed irAEs under treatment with ICIs, including anti-PD-L1, anti-PD-1, anti-CTLA-4, combination ICI, with or without combination with non-immune chemotherapeutics or targeted therapies for either solid or hematologic malignancies. Specifically, we aimed to investigate whether there were differences in outcomes between patients who were rechallenged after an irAE and those who experienced no irAEs or those who had ICIs discontinued. IrAE and ICI-specific characteristics were also evaluated.

## 2. Methods

### 2.1. Study Design and Participants

This retrospective, single center study was approved by the Institutional Review Board of West Virginia University. Patients with active malignancies treated with immune checkpoint monotherapy anti-PD-1/PD-L1 antibodies (e.g., pembrolizumab, nivolumab, durvalumab, atezolizumab, or avelumab) or in combination with anti-CTLA-4 therapy (ipiliumumab), or other established therapies were included. Patients with a histopathologic diagnosis of active malignancy, at least 18 years of age, received at least one cycle of an aforementioned ICI from January 2011 and September 2019 at the West Virginia University Mary Babb Randolph Cancer Center, WVU Cancer Institute (Morgantown, WV, USA) and developed at least one irAE during this time were included in this study. In addition, a matching cohort of 131 patients without an irAE were selected from the database during this timeframe to serve as a comparator arm. Patients with irAEs were dichotomously classified based on subsequent re-exposure to ICI: (1) rechallenged patients had either (a) no interruption in ICI or (b) had reinitiated therapy after interruption; and (2) non-reinitiated patients had no further exposure to ICI after the irAE-related interruption. The definition and grade of an irAE was defined by the Common Terminology Criteria for Adverse Events (CTCAE) v5.0). The data was obtained from the cancer center’s electronic medical record and the data stored in a secure web application, REDCap, via a de-identified, and password-protected fashion.

### 2.2. Procedures

All patients had data collected on demographics, histology, stage at diagnosis, tumor grade, total number of ICI cycles given, total duration on ICI, immunosuppressive therapy for the irAE used, date of diagnosis, date of start of ICI, date of radiologic progression/biopsy proven progression, date of death, disease status after intended ICI completion, substance abuse, Eastern Cooperative Oncology Group performance status, central nervous system (CNS) metastasis status and whether related steroid use was required, steroid or systemic antibiotic use 30 days before ICI initiation, and PD-L1 status per tumor proportion scoring. Disease status was assessed as per the response evaluation criteria in solid tumors (RECIST) 1.0 criteria. Comorbid conditions were defined as obesity (BMI ≥ 30), hypertension, diabetes mellitus, congestive heart failure, chronic obstructive pulmonary disease, any autoimmune related conditions, coronary artery disease, cirrhosis/liver disease, chronic kidney disease, and other non-skin malignancies.

Patients who developed an irAE had the following data obtained: need for immunosuppressive treatment as related to irAE, type of immunosuppressive therapies, type of irAE and its grade, identity of immunotherapy used on rechallenge, date of any second or third recurrent irAE, date of initial and subsequent ICI resumption, and grade of second or third recurrent irAE. Patients were stratified by irAE status and ICI rechallenge status (rechallenged with interruption, rechallenged without interruption, and therapy discontinued without reinitiation post-interruption) following irAE.

### 2.3. Outcomes

Duration from irAE to ICI resumption was defined as the median days from irAE to the first date of ICI resumption. Median observed period was defined as the median days from the first ICI cycle to the cut off period of 30 September 2019. Median duration of initial ICI therapy was defined as the median days from initial ICI cycle to the last cycle prior to an irAE. Median duration of total ICI therapy was defined as the median days from initial ICI therapy to the last ICI cycle administered. Overall survival was defined from time of ICI initiation to death from any cause or censored at last follow-up by 30 September 2019. Response evaluation was investigator-assessed using response evaluation criteria in solid tumors principles (RECIST v1.1).

### 2.4. Statistical Analysis

Patient and disease characteristics were summarized as median and range for continuous variables, and as numerical values/frequencies for categorical variables. The characteristics of patients and initial irAEs were compared between the reinitiated and non-reinitiated groups by Fisher’s Exact test for categorical variables and Wilcoxon’s Rank Sum test for continuous variables. The Cox multivariate proportional analysis model was used to analyze the hazard ratio and effect of checkpoint inhibitor reinitiation status on overall survival, adjusting for covariates. A time to event analysis was incorporated. Comparisons of medians were carried out using the Wald test, and differences with *p* ≤ 0.05 values were regarded as statistically significant. The distribution of overall survival was evaluated with the Kaplan–Meier methodology. The statistical analysis was performed using SAS^®^ software (Version 9.4, SAS Institute Inc., Cary, NC, USA).

## 3. Results

A total of 548 patients who received ICI therapy were screened for study inclusion; 14 patients were excluded due to being lost to follow-up (*n* = 13) and less than 18 years of age (*n* = 1). We identified 133 (25%) patients who developed at least 1 irAE of any grade and a matching 131 patients (25%) cohort that did not; demographics and clinicopathologic characteristics are shown in Table 1. The median age was 65 years (IQR, 59–73 years) versus 63 years (IQR, 55–69 years). Most patients had an ECOG of 0–1 in both cohorts (81% versus 77%). Lung was the most common malignancy type (40% versus 45%) followed by melanoma (29% versus 19%) and genitourinary (11% vs. 10%). CNS disease was present in 35 patients (26%) versus 42 patients (32%), of which 31 patients (89%) versus 34 patients (76%) required palliative steroid treatment.

### 3.1. Initial ICI in Patients with an irAE

The median observation period was 14.5 months (IQR, 6–25) versus 13.3 months (IQR, 4–23). Initial ICI therapy was a PD-1 inhibitor in 87 patients (65%) versus 109 patients (83%), a CTLA-4 inhibitor in 10 patients (8%) versus 9 patients (7%), a combination in 20 patients (15%) versus 1 patient (1%), and a PD-L1 inhibitor in 16 patients (12%) versus 12 patients (9%). 33 patients (25%) versus 28 patients (21%) had steroid use within 30 days of ICI initiation. 31 patients (23%) versus 27 patients (21%) required antibiotic use within 30 days of ICI initiation. 32% of evaluable patients with irAE had a PD-L1 status ≥50% compared to 21% without an irAE.

### 3.2. Initial and Sequential irAEs

The median duration from initial irAE to ICI reinitiation was 28 days (IQR, 16–44; Table 2). 70 patients (61%) developed grade 2 irAE and 45 patients developed grade 3 or 4 irAE (34%). 98 (74%) patients were rechallenged (with or without interruption) with further ICI after initial irAE. The same ICI was used for 83 patients (85%) upon reinitiation, of which 79 patients were administered an anti-PD1 inhibitor. The median duration from ICI reinitiation to 2nd irAE was 3 months (IQR, 1–6). 41 patients (42%) developed a second irAE, a majority being grade 1–2 (71%). Only 11 (27%) patients with a second irAE had the same irAE upon rechallenge. The median duration from initial ICI reinitiation to 3rd irAE was 10 months (IQR, 6–18). 7 patients (5%) developed a third irAE, of which 4 patients developed the same grade and type of irAE as either of the first two irAE. Out of 80 patients (60%) that required immunosuppressive therapies, a majority (79%) were treated with oral or intravenous corticosteroids.

The three most common irAEs after the initial rechallenge were thyroid dysfunction, diarrhea/colitis, and rash (Supplemental Table S1). The least common irAEs included nephritis and myalgia. In patients who had an irAE and were rechallenged with/without ICI interruption, the median duration of initial irAE was documented to be a median of 2 months (IQR 1–4) and occurred sooner than patients with therapy discontinuation without reinitiation (median, 3.5 months; IQR 2–8 months; *p* = 0.02). There were no statistical differences in the median observation period between the two groups (Table 3). The grade of initial irAE were more severe in the non-reinitiated group (*p* < 0.001) and required a greater proportion of patients to be treated with immunosuppression (51% in the rechallenged versus 86% in non-reinitiated, *p* < 0.0001). The median overall survival was 10.1 months (IQR, 6.5–13.6) for patients who did not have an irAE compared to 37.8 months (IQR, 19.7–51.7) in those that did have an irAE and had further therapy reinitiated (HR, 0.38; *p* < 0.0001). The overall survival difference for patients with an irAE between the “ICI rechallenged” (37.8 months) and “ICI interrupted and not reinitiated (i.e., discontinued)” groups (24.9 months) was not found to be significantly significant (*p* = 0.7046).

**Figure 1:**
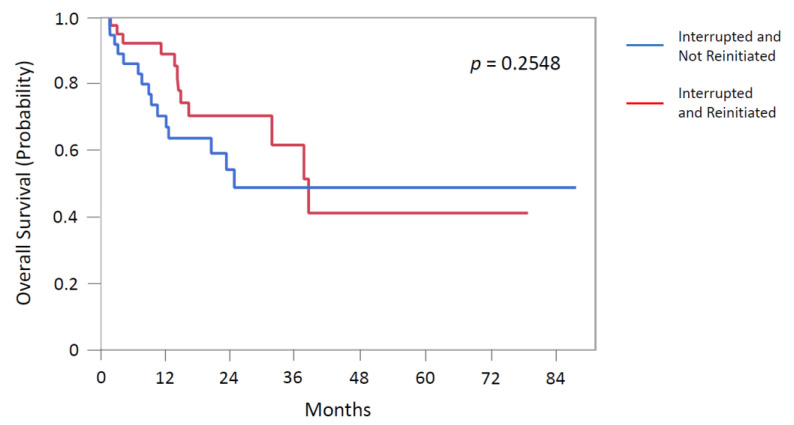
Kaplan-Meier Survival Analysis of Study Patients with ICI irAEs Having ICI Interrupted and Not Reinitiated (i.e., Discontinued) (blue) versus Interrupted and Reinitiated (red).

In patients with ICI interrupted then reinitiated, the median duration of initial irAE was found to be a median of 2 months (IQR 1–4) and occurred sooner than ICI interrupted/discontinued and not reinitiated patients (median, 3.5 months; IQR 2–8; *p* = 0.0876) (Table 4). There were no statistical differences in the median observation period between the two groups. The grades of initial irAE were more serious in the non-reinitiated group (*p* < 0.001), having 80% with grades 3–4 irAEs vs. only 44% in the reinitiated group. Yet similar proportions of patients were found to have been treated with immunosuppression (87% in the reinitiated versus 86% in non-reinitiated, *p* = 0.50) between the two cohorts. The median overall survival was 38.6 months (IQR, 16.4-not reached) for patients who were reinitiated after irAE interrupted period compared to 24.9 months in those who were interrupted but not reinitiated on ICI (i.e., ICI discontinued) (IQR, 12.2-not reached, *p* = 0.2548). Figure 1 depicts the comparison in Kaplan–Meier survival analysis between these two cohorts.

A Cox multivariate proportional model (Table 5) analysis of the irAE positive patients demonstrates that the ICI “rechallenged post-interruption” cohort (interrupted and then reinitiated group) is not significantly associated with lower risk of death (HR = 1.19; CI 0.70–2.03; *p* = 0.52) compared to the ICI “not rechallenged” cohort (interrupted and non-reinitiated), adjusted for ICI duration of use. Patients treated with anti-PD-L1 ICI were associated with a higher risk of death (HR = 2.67, CI 1.19–6.00; *p* = 0.02). Being treated with anti-CTLA-4-based ICI regimen was associated with lower risk of death compared to anti-PD-1 (HR = 0.50, CI 0.26–0.94; *p* = 0.03). ECOG performance status and tumor locations were not significant covariates. Of note, the irAE grades were not found to be significant covariates. Supplemental Figure S1 shows the Kaplan–Meier survival analysis of ICI “interrupted and non-reinitiated” versus the “no irAEs” group (*p* < 0.0001).

## 4. Discussion

To our knowledge, this is one of the first real-world experience studies with long-term follow-up conducted at a single center that investigated the survival outcomes in patients with irAEs who were rechallenged with immunotherapy to treat solid or hematologic malignancies. In our current study, a Cox multivariate proportional analysis adjusted for time to events, found that among patients who developed irAEs and also with ICI therapy interrupted, those who subsequently had ICI therapy reinitiated did not have statistically significantly lower risk of death (HR, 1.19; *p* = 0.52) than patients who did not have ICI therapy reinitiated after interruption, despite a longer median ICI treatment duration in the former. These cohort groups were well balanced in demographics.

Our study also demonstrates that cancer patients who developed any irAEs and were rechallenged with ICI (with or without ICI treatment interruption) had significantly longer overall survival (HR, 0.34; *p* < 0.0001) than patients who did not develop irAEs. (Supplemental Table S2; Supplemental Figure S2) While there was a trend towards an increase in co-morbidities in the non-irAE group, the performance status was not different between these two groups. These findings corroborate well with the conclusions from several previous studies that focused on non-small cell lung cancer and melanoma [6,12]. Interestingly, a recent study has suggested a correlation between grade 3 or 4 immunotherapy related adverse events (irAEs) and the degree of durable response. Patients with advanced melanoma treated with ipilimumab who experienced grade 3 toxicities requiring steroids were found to have higher response rates to therapy and a longer median duration of response [13]. It was postulated by the investigators that these toxicities may reflect increased immunotherapy activity in melanoma, and thus as a result explaining the improved patient outcomes. Factors that predict irAEs, such as history of autoimmune disease, use of CTLA-4 inhibitors, and poor kidney function of grade 3 or higher, have been investigated and reported to date [3]. Within each malignancy, there can be found an abundance of intra-tumor heterogeneity and response/evolution to therapy [14]. This may explain the predominant findings that melanoma and lung cancer patients who developed irAE are the ones also appeared to have improved overall survival compared to patients who did not develop irAE. A recent multicenter study further identified that development of multisystem irAEs was associated with improved survival outcomes in patients with advanced NSCLC treated ICIs [15]. In this study with multivariate model analysis, patients with 1 irAE and multisystem irAEs showed incrementally improved overall survival (adjusted HR, 0.86; *p* = 0.26; and adjusted HR 0.57; *p* = 0.005, respectively) compared with patients with no irAEs. Emerging evidence from these studies lend a growing support to the notion that irAEs under ICI cancer immunotherapy may be reflective of the mechanism-based autoimmune or inflammatory reactions towards the ICI. Hence, it could be a manifestation of the host’s systemic immune response primed and activated under the ICI therapy, and thus could in turn be predictive of more favorable clinical outcomes, given that the irAEs are managed appropriately.

Hyperthyroidism and hypothyroidism were the most common irAE observed in our patient population. PD-1 inhibitors are generally believed to inhibit T cells at later stages of the immune response in peripheral tissues and may improve survival outcomes in patients undergoing ICI treatment [16,17]. Interestingly, multivariable analysis in our study suggested improved outcomes in patients who had either anti-CTLA-4 ICI alone or with combination anti-PD-1 ICI. Conversely, anti-PD-L1 ICI was associated with worsened outcomes compared to anti-PD-1 ICI and this finding is corroborated by the aforementioned meta-analysis of 19 randomized clinical trials [17]. In addition to a class effect, there is a growing belief that the microbiologic composition of a patient’s gastrointestinal flora is associated with irAE development [18]. Our study did not demonstrate a survival difference in patients who had antibiotic use within 30 days of ICI initiation compared with those who did not. However, there is growing literature that suggests antibiotic use in this period adversely affects survival, although we were unable to demonstrate this in our study [19].

In one multivariate analysis of a pooled cohort of patients, the use of baseline corticosteroids at >10 mg/day of prednisone or its equivalence was independently associated with worse PFS and OS [20]. However, ours and other studies in NSCLC did not find this same association [21]. With regards to immunotherapy pharmacokinetics, it is currently accepted that there is a reduction in clearance of these therapies over time. Immunotherapeutic plasma levels have been noted to increase over time [22]. Factors such as larger baseline tumor size, lower Eastern Cooperative Oncology Group performance status score, and higher tumor response to treatment may potentially be associated with this reduced clearance and subsequent improved patient outcomes [23]. The potential confounding variable of corticosteroid use prior to the course of immunotherapy may be dose independent. However, one study suggested that the discontinuation of such therapy before the first dose of anti-PD-1 inhibitor was administered led to patient outcomes similar to corticosteroid-naïve patients [23]. In vivo studies of T-cells in ICI therapy have shown that CTLA-4, but not PD-L1, blockade can partially prevent the inhibitory effects of corticosteroids on the immune system and may partially explain the class effect demonstrated above [24]. The use of steroids before ICI initiation was not associated with reduced OS in our current study.

The decision of whether to rechallenge upon an irAE is an important and practical dilemma of growing interest, with an emphasis and need to accomplish in a clinically safe manner [11,25]. A 27% recurrence rate of the initial irAE was observed in our study compared to 28.8% in a recent large cross-sectional cohort study [26]. Two of the more common recurrent irAEs were colitis and pneumonitis, similar to other large studies [26]. Our study did not demonstrate a statistically significant survival difference between the “interrupted and reinitiated” versus “interrupted and non-reinitiated” patients after at least one irAE occurrence on ICI (Figure 1, Table 5). Notably, Cox multivariate proportional analysis in our study did not identify the severity of irAE grades as significant covariates. In a recent multivariate analysis of anti-PD-1-induced irAEs and survival outcomes in advanced melanoma, development of grade ≥3 irAEs (HR, 0.29, *p* = 0.024) was significantly associated with longer OS [12]. We acknowledge that our study analysis finding above could possibly be a result of our relatively small cohort size limitation and resultant lack of power. Nonetheless, we note that our study cohorts sample size is quite comparable with the recent related studies in this important topic including multicenter and global cohort studies [12,15,27]. Moreover, our study results could have significant implication and impact on clinical practice in ICI management in the context of treatment decisions regarding ICI “rechallenge or not” especially after ICI interruption due to irAEs. The recent study by Naqash et al. demonstrated a negative impact of irAE-related treatment discontinuation on survival; however, the exploratory study focused only on the use of nivolumab in NSCLC 2nd or further lines of therapy [27]. Currently, effort is underway to extend our single center study into a multicenter collaborative study to increase the study cohort size for validation and a better powered analysis.

There are several limitations to our study. First, the study was conducted at a single institution in a state with generally higher medical comorbidities and reported cancer disparities compared to the rest of the country. Thus, generalizability to the general national population may be limited. Furthermore, there may be intrinsic cancer biology and molecular landscape differences in the tumors of Appalachian cancer patients due to unique geographic, social, cultural and epidemiologic variances of the population. Second, this is a retrospective study that has an expectant intrinsic selection bias. Third, the sample size was relatively modest and limited our ability to analyze subgroups within the irAE group. Although our cohort incorporated multiple cancer subtypes, multivariable analysis suggested that the survival benefit in the rechallenged group also applied to the largest cohort groups (lung, melanoma). Lastly, the decision to rechallenge with or without ICI discontinuation in the face of irAEs in this study was primarily clinician dependent, guided by individual clinical judgement and national guidelines (e.g., NCCN), and thus a potential confounding variable. Nonetheless, it also highlights the urgent need for further outcome research into the critical questions we posed in our study relating to the many levels of clinical dilemma and treatment decisions in the face of significant irAEs under ICI therapy, especially after treatment interruption.

## 5. Conclusions

Manifestation of irAEs onset is a common event under ICI cancer therapy; and appears to be a favorable predictive and prognostic marker based on emerging literature. Hence, it is a clinically relevant and pressing question whether patients should be rechallenged with ICI in the event of irAEs occurrence, with or without ICI treatment interruption, in order to maximize ICI clinical benefits. Here, our single center retrospective study findings identified no significant improvement in survival outcomes among the ICI-treated patients who developed irAEs, and resultant ICI therapy interruption followed by reinitiation, compared with those with ICI non-reinitiation. Hence reinitiation of ICI after irAEs-related treatment interruption may not correlate with improved survival outcomes and may not always be clinically warranted. Further research is urgently needed to explore the relationship between initiation of immunotherapy, subsequent immune-mediated toxicities within the inflammatory micro/macro-environment, extent of durable response in patients that respond to such therapies, and ultimately patient outcomes after ICI rechallenge in all malignancy types.

## Figures and Tables

**Table 1:** General Characteristics of the Study Patients who had ICI Treatment With and Without irAE.

Characteristics	Patients, No. (%)		
	Patients with irAE (N = 133)	Patients without irAE (N = 131)	*p*
Median age, years (IQR)	65 (59–73)	63 (55–69)	0.07
Male Sex	71 (53)	80 (61)	0.21
Caucasian	131 (99)	124 (95)	0.13
Comorbid conditions present	91 (68)	106 (81)	0.08
ECOG Performance Status			
0–1	108 (81)	101 (77)	0.45
2–3	25 (19)	30 (23)	0.44
Tumor Grade			
Poorly differentiated	66 (50)	78 (60)	0.10
Cancer Stage	N = 114	N = 118	
III	27 (20)	28 (21)	0.83
IV	87 (65)	90 (69)	0.57
Cancer Type			
Lung	53 (40)	59 (45)	0.39
Melanoma	39 (29)	25 (19)	0.04
Genitourinary	14 (11)	13 (10)	0.87
Other solid *	9 (1)	4 (3)	0.57
Hematologic **	3 (2)	4 (3)	0.61
CNS disease present	35 (26)	42 (32)	0.30
Steroids required for CNS disease	31 (89)	34 (76)	0.51
Median observation period, months (IQR)	14.5 (6–25)	13.3 (4–23)	
Initial Immunotherapy			
Anti-PD-1	87 (65)	109 (83)	0.45
Anti-CTLA-4	10 (8)	9 (7)	0.84
Combination anti-PD-1/CTLA-4	20 (15)	1 (1)	<0.001
Anti-PD-L1	16 (12)	12 (9)	0.05
Steroid use within 30 days of ICI initiation	33 (25)	28 (21)	0.51
Antibiotic use within 30 days of ICI initiation	31 (23)	27 (21)	0.60
PD-L1 Status (% TPS)	N = 65	N = 57	
≥50	21 (32)	12 (21)	0.04
1–49	19 (29)	24 (42)	0.03
<1	25 (38)	21 (37)	0.9

Abbreviations: ECOG, eastern cooperative oncology group; CNS, central nervous system; IQR, interquartile range; ICI, immune checkpoint inhibitor; irAE, immune related adverse event; PD-1/L1, programmed cell death-1 or ligand 1; CTLA-4, cytotoxic T-cell lymphocyte-4; * Other solid types include: duodenal, breast, head and neck, adrenal cortical carcinoma, esophageal, colon, ovarian, gastric, merkel cell, endometrial, and cervical cancer. PD-L1 status was measured by tumor proportion score (TPS, %). ** Hematologic malignancies include: Hodgkin’s lymphoma, mycosis fungoides, and diffuse large B cell lymphoma.

**Table 2:** Characteristics of Initial and Sequential irAEs.

irAE (*n* = 133)	Patients, No. (%)
Hypothyroidism	43 (32)
Rash	30 (23)
Diarrhea/colitis	22 (17)
Pneumonitis	17 (13)
Adrenal Insufficiency	12 (9)
Hepatitis	9 (7)
Nephritis	6 (5)
Hypophysitis	3 (2)
Diabetes mellitus	3 (2)
Myalgia	3 (2)
Encephalopathy	2 (2)
Other **	8 (6)
Grade of irAE (*n* = 115)	
2	70 (61)
3–4	45 (39)
Immunotherapy rechallenged after irAE	98 (74)
Immunotherapy interrupted (with or without subsequent reinitiation) after irAE	74 (56)
Identical Immunotherapy used after irAE (*n* = 98)	83 (85)
Immunotherapy used after irAE (*n* = 98)	
Anti-PD-1	79 (81)
Anti-CTLA-4	2 (2)
Combination anti-PD-1/CTLA-4	5 (5)
Anti-PD-L1	12 (12)
Median duration of ICI therapy after irAE, cycles (IQR)	7 (3–14.75)
Median duration from irAE to ICI resumption, days (IQR, *n* = 38)	28 (16–44)
Median duration from ICI resumption to 2nd irAE, months (IQR, *n* = 38)	3 (1–6)
Median duration from ICI resumption to 3rd irAE, months (IQR, *n* = 7)	10 (6–18)
New IrAE after reinitiation of Immunotherapy (*n* = 98)	41 (42)
Grade of second irAE (*n* = 41)	
1–2	29 (71)
3–4	12 (29)
IrAE after ICI interruption and reinitiated rechallenge the same as first irAE	11 (27)
IrAE after second interruption and reinitiated rechallenge (*n* = 7) the same as first irAE	4 (57)
Grade of irAE after second reinitiation (*n* = 7)	
1–2	4 (57)
3	3 (43)
Need for Immunosuppression	80 (60)
PO/IV Steroids	63 (79)
TNF-alpha inhibition	3 (<1)
Topical steroids	14 (18)

** Other irAE included arthralgia, MAHA, uveitis, SIADH, aseptic meningitis.

**Table 3:** “Rechallenged (Interrupted and Reinitiated + Not interrupted and Reinitiated after irAE)” compared to “Discontinued and Not Reinitiated”.

Characteristics	ICI Rechallenged Patients, No. (%)	ICI Discontinued and Not Reinitiated Patients, No. (%)	*p*
No. of patients	98	35	
Age, IQR	64 (58.25–72)	68 (60–74)	0.10
Alcohol status			
Current	27 (28)	6 (17)	0.56
Former	32 (33)	15 (43)	
Smoking status			
Current	16 (16)	7 (20)	0.28
Former	53 (54)	22 (63)	
Never	29 (30)	6 (17)	
Comorbid conditions present	71 (72)	20 (57)	0.10
ECOG Performance Status			
0–1	84 (86)	29 (83)	0.75
2–3	14 (14)	6 (17)	
Median observed period, days	434.5 (173.5–771.5)	446 (222–707)	0.81
Median duration of ICI therapy, months (IQR)	10 (4–17)	3 (1–6)	<0.0001
Median duration between initial ICI therapy and initial irAE, months (IQR)	2 (1–4)	3.5 (2–8)	0.02
Initial Immunotherapy			
Anti-PD-1	65 (66)	22 (63)	0.97
Anti-CTLA-4	7 (7)	3 (9)	
Combination anti-PD-1/CTLA-4	15 (15)	5 (14)	
Anti-PD-L1	11 (11)	5 (14)	
Steroid use within 30 days of ICI initiation	23 (23)	10 (29)	0.98
Antibiotic use within 30 days of ICI initiation	24 (24)	7 (20)	0.26
CNS disease present	26 (27)	9 (26)	0.82
Steroids used for CNS disease	23 (23)	8 (23)	0.98
PD-L1 Status (% TPS)	48	17	
≥50	15 (31)	6 (35)	0.32
1–49	15 (31)	4 (11)	
<1	18 (38)	7 (20)	
Grade of irAE			
2	63 (64)	7 (20)	<0.0001
3–4	17 (17)	28 (80)	
Median duration of ICI therapy prior to irAE, cycles (IQR)	3 (2–6)	4 (3–8.5)	0.03
Need for Immunosuppression	50 (51)	30 (86)	<0.0001
PO/IV Steroids	34 (67)	29 (97)	
TNF-alpha inhibition	2 (4)	1 (3)	
Topical steroids	14 (28)	0 (0)	
Disease status after completion of ICI	96	33	
Complete response	27 (28)	10 (30)	0.80
Partial response	10 (10)	2 (6)	
Stable disease	17 (18)	8 (24)	
Progression of disease	42 (44)	13 (39)	
mOS, months (IQR)	37.8 (19.7–51.7)	24.9 (12.2-NR)	0.7046

**Table 4:** Characteristics of Patients with IrAE and Stratified by ICI Interrupted Status.

Characteristics	ICI Interrupted, then Reinitiated Patients, No. (%)	ICI Interrupted and Not Reinitiated (i.e., Discontinued) Patients, No. (%)	*p*
No. of patients	39	35	
Age, IQR	64 (59–71.5)	68 (60–74)	0.91
Alcohol status			
Current	10 (26)	6 (17)	0.52
Former	11 (28)	15 (43)	
Smoking status			
Current	7 (18)	7 (20)	0.60
Former	22 (56)	22 (63)	
Never	10 (26)	6 (17)	
Comorbid conditions present	27 (69)	20 (57)	
ECOG Performance status			
0–1	35 (90)	29 (83)	0.44
2–3	4 (10)	6 (17)	
Median observed period, months (IQR)	16 (11–31)	15 (7–24)	0.38
Median duration of total ICI therapy, months (IQR)	12 (4–21.5)	3 (1–8)	<0.001
Median duration between initial ICI therapy and initial irAE, months (IQR)	2 (1–4)	3.5 (2–8)	0.09
Initial Immunotherapy			
Anti-PD-1	20 (51)	22 (63)	0.75
Anti-CTLA-4	5 (13)	3 (9)	
Combination anti-PD-1/CTLA-4	8 (21)	5 (14)	
Anti-PD-L1	6 (15)	5 (14)	0.58
Steroid use within 30 days of ICI initiation	8 (21)	10 (29)	0.90
Antibiotic use within 30 days of ICI initiation	11 (28)	7 (20)	0.13
CNS disease present	15 (38)	9 (26)	0.40
Steroids used for CNS disease	13 (33)	8 (23)	0.68
PD-L1 Status	*n* = 48	*n* = 17	
≥50	6 (15)	6 (35)	0.58
1–49	6 (15)	4 (11)	
<1	8 (21)	7 (20)	
Grade of irAE			
2	19 (49)	7 (20)	<0.001
3–4	17 (44)	28 (80)	
Median duration of ICI therapy prior to irAE, cycles (IQR)	4 (2–6)	4 (3–8.5)	
Need for Immunosuppression for irAE	*n* = 34 (87)	N = 30 (86)	0.50
PO/IV Steroids	28 (82)	29 (97)	
TNF-alpha inhibition	2 (6)	1 (3)	
Topical steroids	4 (12)	0 (0)	
Disease status after completion of ICI	39	33	
Complete response	11 (31)	10 (30)	
Partial response	5 (14)	2 (6)	
Stable disease	8 (22)	8 (24)	
Progression of disease	15 (33)	13 (39)	
mOS, months (IQR)	38.6 (16.4-NR)	24.9 (12.2-NR)	0.2548

**Table 5:** Cox multivariate proportional model depicting the risk of overall survival in groups of ICI reinitiation status adjusting for the effect of covariates.

Effect	Categories	Hazard Ratio *	Lower CI	Upper CI	*p*
Group	IrAE and not rechallenged (interrupted + non-reinitiated)	Ref			
	IrAE and rechallenged post-interruption (interrupted + reinitiated)	1.19	0.70	2.03	0.52
Age	≤60Y	Ref			
	>60Y	1.59	0.90	2.79	0.11
Gender	Male	Ref			
	Female	0.67	0.39	1.13	0.13
Immunotherapy type	Pembro/Nivo (PD-1)	Ref			
	Ipi & Ipi/Nivo (CTLA-4/PD-1)	0.50	0.26	0.94	0.05
	Atezo/Durva/Avelu (PD-L1)	2.67	1.19	6.00	0.01
Antibiotic use	No	Ref			
	Yes	1.40	0.78	2.49	0.13
IrAE Grade	1–2	Ref			
	3–4	0.93	0.50	1.73	0.83
Median ICI duration, months		0.96	0.94	0.98	0.001

* Ref: Reference for hazard ratio analysis and comparison.

## Data Availability

The data presented in this study are available on request from the corresponding author. The data are not publicly available due to HIPAA concerns.

## Supplementary Materials

The following are available online at https://www.mdpi.com/2072-6694/13/5/989/s1, Table S1: Type of irAEs after Initial ICI Reinitiation. Table S2: Cox multivariate proportional analysis irAE rechallenged vs. non-irAE. Figure S1: Kaplan-Meier Survival Analysis of Study Patients with No irAEs (blue) compared with those ICI “Interrupted and Not Reinitiated (i.e., Discontinued)” (red). Figure S2: Kaplan-Meier Survival Analysis of the Study Patients with No irAEs (blue) compared with the “ICI Rechallenged” Cohort (ICI Reinitiated +/− Interruption) after irAEs.

## Declarations

This manuscript has been read and approved by all the authors, the requirements for authorship have been met, and each author believes that the manuscript represents honest and accurate work worthy of publication. There are no competing interests to this manuscript. All authors had access to the data and material.

## Abbreviations

irAE	immune related adverse event
ICI	immune checkpoint inhibitor
mOS	median overall survival
PD-1	programmed death 1
PD-L1	programmed death-ligand 1
CTLA-4	cytotoxic T-lymphocyte –associated protein 4
CNS	central nervous system
RECIST	Response Evaluation Criteria In Solid Tumors
CTCAE	Common Terminology Criteria for Adverse Events
TPS	tumor proportion score
NCCN	National Comprehensive Cancer Network
