# Supplementary material for: Immune-Related Adverse Events (irAE) in Cancer Immune Checkpoint Inhibitors (ICI) and Survival Outcomes Correlation: To Rechallenge or Not?
Source: Cancers (Basel). 2021 Feb 27;13(5):989. doi: 10.3390/cancers13050989 (PMC7956829; doi:10.3390/cancers13050989)
Supplement: Supplementary file 1 [file cancers-13-00989-s001.pdf]

Immune-Related Adverse Events (irAE) in Cancer Immune Checkpoint Inhibitors (ICI) and Survival Outcomes Correlation: To Rechallenge or Not?

Heidar J. Albandar, Jacob Fuqua, Jasim M. Albandar, Salahuddin Safi, Samuel A. Merrill and Patrick C. Ma

Table S1. Type of irAEs after Initial ICI Reinitiation.

**IrAE (*n* = 41)**	***n* (%)**
Hypothyroidism/hyperthyroidism	9 (22)
Diarrhea/colitis	8 (20)
Rash	6 (15)
Pneumonitis	5 (12)
Adrenal Insufficiency	4 (10)
Hepatitis	4 (10)
Nephritis	1 (2)
Myalgia	1 (2)
Other **	3 (7)

** Other irAE included arthralgia, MAHA, and SIADH.

Table S2. Multivariable Cox Proportion Analysis of Overall Survival Comparing irAEs with or without Rechallenge vs Non-irAE Groups.

**Effect**	**Categories**	**Hazard Ratio ***	**Lower CI**	**Upper CI**	***p***
Group	No immune-related adverse events	Ref			
	IrAE and not rechallenged (non-reinitiated + interruption)	0.40	0.22	0.52	0.0024
	IrAE and rechallenged (reinitiated +/- interruption)	0.34	0.22	0.52	<0.0001
Age	≤60Y	Ref			
	>60Y	1.48	1.01	2.17	0.04
Gender	Female	Ref			
	Male	0.77	0.53	1.11	0.16
Immunotherapy type	Pembro/Nivo	Ref			
	Ipi & Ipi/Nivo	1.89	0.88	3.47	0.1
	Atezo/Durva/Avelu	0.98	0.47	2.04	0.95
Antibiotic use	No	Ref			
	Yes	0.76	0.49	1.20	0.24
ECOG Performance status	0–1	Ref			
	>1	2.00	1.35	2.98	0.0006
Tumor Location	Lung	Ref			
	Melanoma	0.31	0.16	0.62	0.0008
	Other	0.84	0.55	1.28	0.41

* Ref: Reference for hazard ratio analysis and comparison.

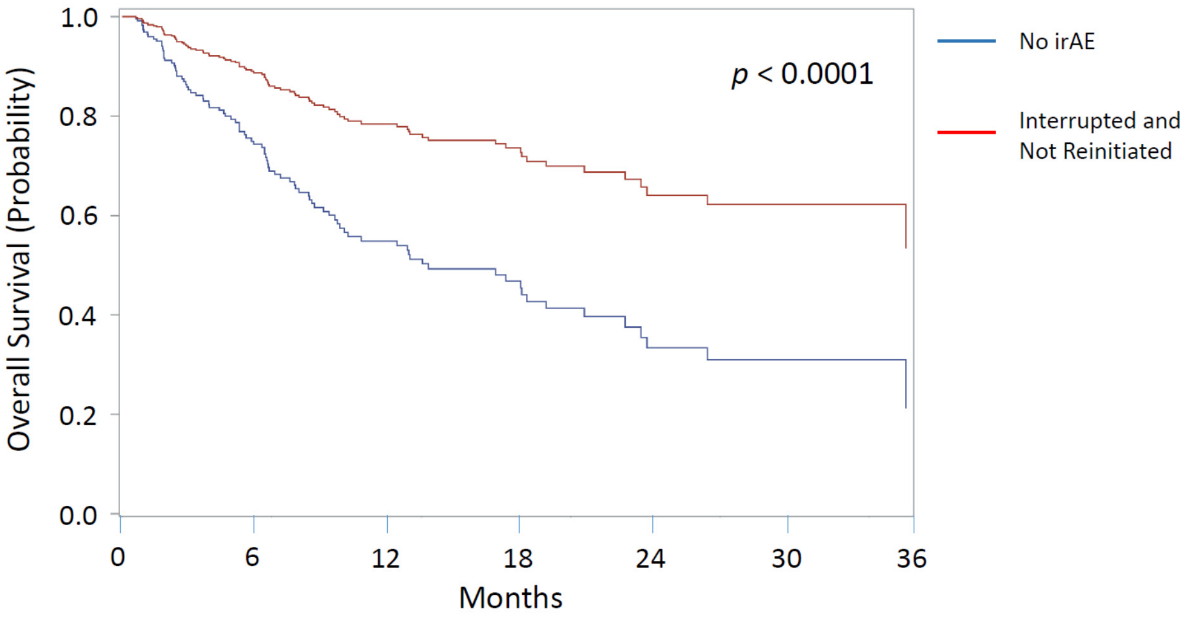

Figure S1. Kaplan-Meier Survival Analysis of Study Patients with No irAEs (blue) compared with those ICI "Interrupted and Not Reinitiated (i.e., Discontinued)" (red).

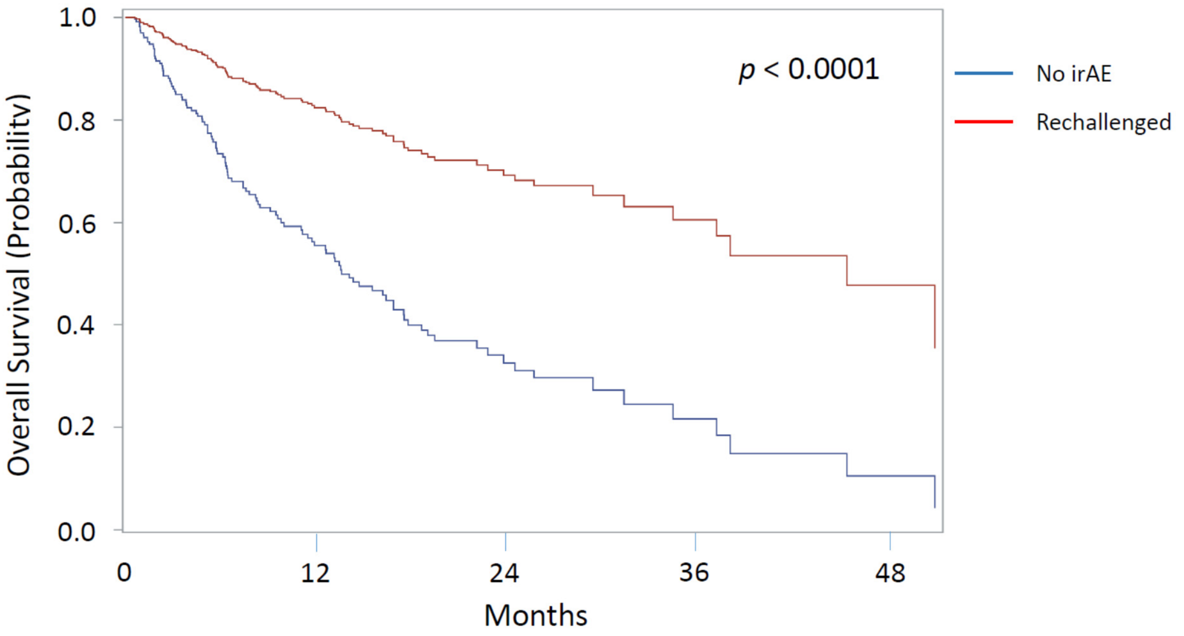

Figure S2. Kaplan-Meier Survival Analysis of the Study Patients with No irAEs (blue) compared with the "ICI Rechallenged" Cohort (ICI Reinitiated +/- Interruption) after irAEs.
